# Topical application of the cold‐mimetic l‐menthol decreases wheel running without affecting the beneficial effects of voluntary exercise in mice

**DOI:** 10.1113/EP092754

**Published:** 2025-06-11

**Authors:** Annalaura Bellucci, Bradley J. Baranowski, Stewart Jeromson, Michael Akcan, Serena Trang, Meagan Arbeau, Hadil Alfares, Katelyn Eisner, David C. Wright

**Affiliations:** ^1^ School of Kinesiology University of British Columbia Vancouver British Columbia Canada; ^2^ British Columbia Children's Hospital Research Institute Vancouver British Columbia Canada; ^3^ Faculty of Land and Food System University of British Columbia Vancouver British Columbia Canada

**Keywords:** exercise, menthol, mice, thermogenesis, thermoneutral, TRPM8

## Abstract

Topical application of l‐menthol, a pharmacological cold‐mimetic and agonist of the cold‐sensing receptor TRPM8 (transient receptor potential cation channel subfamily M member 8), has been shown to stimulate brown adipose tissue (BAT) thermogenesis and reduce weight gain in both obese and lean male mice, without affecting energy intake. While these findings suggest that l‐menthol could offer a novel approach to prevent weight gain, its potential to enhance the benefits of exercise on whole‐body metabolic health remains unexplored. In this study, we investigated whether daily topical l‐menthol application, combined with voluntary wheel running, could enhance exercise‐induced improvements in metabolic health in male and female C57BL/6J mice housed at thermoneutrality (29°C). Our results demonstrated that although l‐menthol treatment reduced voluntary wheel running distance, there was still a main effect of exercise to reduce fat mass, weight gain and improve glucose tolerance. Indirect calorimetry revealed that l‐menthol increased total energy expenditure, potentially explaining improvements in metabolic health despite reductions in voluntary wheel running. These findings suggest that although l‐menthol does not enhance the effects of voluntary exercise, it remains a promising strategy for improving metabolic health.

## Abstract (highlights)

- **What is the central question of this study?**
  Are there beneficial combined effects of topical menthol treatment and voluntary physical activity on indices of metabolic health in mice?
- **What is the main finding and importance?**
  Despite reducing wheel running activity, menthol treatment did not impair the ability of exercise to prevent weight gain and improve glucose homeostasis. Menthol treatment increased energy expenditure, which might explain why the effects of exercise were maintained despite reductions in running activity. These findings are important as they highlight interactions between exercise and menthol on improving metabolic health.

## 1 INTRODUCTION

Transient receptor potential (TRP) channels, a family of thermoreceptors, are primarily expressed in afferent dorsal root ganglion (DRG) sensory neurons (Bautista et al., 2007) and trigeminal ganglia that innervate the skin, functioning to relay environmental temperature changes to the central nervous system (CNS) (Clapham, 2003). Among these channels, the TRP cation channel subfamily M member 8 (TRPM8) plays a key role in cold‐sensing through the activation of brown adipose tissue (BAT) thermogenesis (McKemy et al., 2002). TRPM8 is activated by innocuous cooling temperatures below 30°C and is essential for detecting unpleasant cold stimuli or mediating the effects of cold‐induced analgesia (Bharate & Bharate, 2012). Additionally, it serves as a receptor for chemical ligands, such as l‐menthol and icilin, which are naturally derived and synthetic cooling compounds, respectively (Peier et al., 2002). In C57BL/6J male mice housed at thermoneutrality, activation of TRPM8 via topical l‐menthol application has been shown to stimulate BAT thermogenesis, attenuate weight gain and increase energy expenditure through a UCP1 and noradrenaline‐dependent mechanism (McKie et al., 2022). In human studies a single topical application of l‐menthol has been shown to increase thermogenesis (Valente et al., 2015), metabolic rate and exercise performance, particularly in hot environments (Flood, 2018; Flood et al., 2017; Roriz et al., 2024; Stevens & Best, 2017). In young, healthy men, acute oral administration of l‐menthol has been shown to induce hyperventilation, improve endurance capacity and lower the rate of perceived exertion (Mundel & Jones, 2010). Similarly, in well‐trained male runners, l‐menthol ingestion improved breathing comfort and boosted endurance capacity during exhaustive endurance running (Tsutsumi et al., 2023).

Furthermore, a meta‐analysis of randomized control trials found that l‐menthol promoted a cooler thermal sensation and improved thermal comfort during exercise (Keringer et al., 2020). Factors such as environmental conditions, body mass index and route of application have been identified as key contributors to enhancing the effects of l‐menthol on endurance performance in humans (Keringer et al., 2020). Given the promising evidence that topical application of the pharmacological cold‐mimetic l‐menthol mitigates weight gain in rodents and enhances thermogenesis, metabolic rate and exercise performance in humans, it is compelling to explore whether topical l‐menthol application can potentiate the metabolic benefits of exercise. Therefore, the aim of this study was to investigate the impact of topical l‐menthol treatment on the effects of voluntary physical activity in mice. We hypothesized that menthol treatment would potentiate the metabolic effects of voluntary wheel running in mice of both sexes housed at thermoneutrality.

## 2 METHODS

### 2.1 Ethical approval

All protocols were approved by the University of British Columbia Animal Care Committee (protocol no. A22‐0011) and followed Canadian Council on Animal Care Guidelines and adhered to *Experimental Physiology*’s policies regarding animal experimentation.

### 2.2 Animals

Male and female C57BL/6J mice (∼12–14 weeks of age) (cat. no. 000664, 120 female and 32 male mice, The Jackson Laboratory, Bar Harbor, ME, USA) were group‐housed (∼4/cage) at room temperature (20–22°C) and given ad libitum access to standard chow (cat. no. 2918, Teklad, West Lafayette, IN, USA) and water while acclimating to the animal facility at BC Children's Hospital Research Institute for 1 week. Thereafter, mice were single housed in shoe‐box cages (18.4 × 29.2 × 12.7 cm, W × L × H) given ad libitum access to standard chow diet and water and acclimated at thermoneutrality (29°C) in the Solace Zone Caging System (Alternative Design Manufacturing & Supply Inc., Siloam Springs, AR, USA).

### 2.3 Topical l‐menthol treatments and voluntary wheel running

After 1 week of acclimation at thermoneutrality (29°C), 12‐ to 14 week‐old C57BL/6J female and male mice were weight‐matched, given access to a running wheel or remained sedentary for 3 weeks, and treated daily with topical l‐menthol (2 g/kg BW, 5% w/v cat. no. W266590 Sigma‐Aldrich, St Louis, MO, USA) or ethanol control (McKie et al., 2022) (8 mice/group in each sex). l‐Menthol or ethanol was selectively applied to the unshaved dorsal surface of restrained mice carefully avoiding orifices, limbs and the tail, as previously described (McKie et al., 2022). All treatments occurred between 10.00 and 12.00 h. Running distance was recorded daily, and body weight and food intake were measured weekly. Eight mice/group in each sex were used for this experiment. One vehicle‐treated female mouse and three vehicle‐treated male mice in the exercise groups were excluded from the analysis as they did not run (<1 km total over the duration of intervention). Pre‐ and post‐intervention, body composition was measured using an EchoMRI body composition analyser (Echo MRI, Houston, TX, USA). Tissue collections occurred 24 h after the last l‐menthol or ethanol control treatment. Running wheels were locked the night before (∼20.00 h), and at ∼07.00 h mice were anaesthetized with pentobarbital (Euthansol 120 mg/kg BW, i.p.) and euthanized through exsanguination of the heart (Medak et al., 2024). In the second set of experiments, 12‐ to 14‐week‐old C57BL/6J female mice housed at thermoneutrality (29°C) were given access to a running wheel or remained sedentary for 2 weeks and were treated daily with a relative dose (2 g/kg) of topical l‐menthol (5% w/v) or the ethanol control (*n* = 8/group). Two vehicle‐treated mice given access to running wheels did not run, and thus were excluded from the analysis. On day 15 the last l‐menthol or ethanol control treatments occurred between 10.00 and 12.00 h. Mice were anaesthetized with pentobarbital (Euthansol 120 mg/kg BW, i.p.) and euthanized through exsanguination of the heart (Medak et al., 2024), and tissue harvested after 6 h of voluntary wheel running activity in the dark cycle following their peak running activity (∼00.00 h).

#### 2.3.1 Oral glucose tolerance test

Glucose tolerance was assessed in male and female mice from the first set of experiments, ∼24 h after the last topical l‐menthol/ethanol control treatment during the third week of intervention, and running wheels were locked at 18.00 h the day before the oral glucose tolerance test. Mice were fasted for 6 h (starting at ∼07.00 h) and given an oral gavage of glucose (2 g/kg BW) (Jeromson et al., 2024).

Blood glucose was taken from a small drop of blood from the tail vein of restrained, conscious mice, using a handheld glucometer and glucose strips (Freestyle Lite; Abbott Laboratories, Abbott Park, IL, USA) and measured immediately pre‐gavage (time point 0) as well as 15, 30, 60, 90 and 120 min post‐gavage.

### 2.4 Acute topical l‐menthol treatment and forced treadmill running

Twelve‐ to fourteen‐week‐old C57BL/6J female mice (*n* = 10/group) housed at thermoneutrality (29°C) were weight‐matched and assigned to either l‐menthol or ethanol control and exercised or sedentary groups. Mice in the exercise groups were familiarized to a motorized rodent treadmill (Exer 3/6, Columbus Instruments, Columbus, OH, USA) for two consecutive days for 15 min/day at 15 m/min on a 5% incline and then given a rest period of ∼48 h before maximum running speed was assessed as previously described (Arbeau et al., 2024; Townsend et al., 2022). Maximum speed was determined by running mice at 10 m/min for 3 min at a 5% incline, and the speed was subsequently increased by 3 m/min every 3 min. Maximum speed was defined as the fastest speed mice could maintain for 3 continuous minutes. Approximately 48 h following the maximum speed test, mice received an acute topical l‐menthol or ethanol control treatment between 10.00 and 12.00 h. The exercise groups ran for 2 h in the dark cycle (start ∼18.00 h), or until exhaustion, at 70% of their predetermined maximum speed with an incline of 5%. The sedentary mice were in the same procedure room as the exercised mice when conducting treadmill acclimation, maximum speed test and 2 h of acute treadmill running.

#### 2.4.1 Indirect calorimetry measurements

Prior to the beginning of the metabolic cage measurements, 12‐ to 14‐week‐old C57BL/6J female mice, housed at thermoneutrality (29°C), were treated daily with either a topical application of l‐menthol (2 g/kg) or an ethanol control (*n* = 8/group). After a 2‐week treatment period, mice were individually housed in metabolic cages (Promethion High‐Definition Multiplexed Respirometry System for Mice; Sable Systems International, Las Vegas, NV, USA) at thermoneutrality (29°C), with ad libitum access to food and water. Mice were allowed to acclimate for 24 h before collecting data over a full light and dark cycle. Mice continued receiving their respective daily treatments of either l‐menthol or ethanol throughout the duration.

Respirometry values were recorded every 5 min, with a 30‐s dwell time per cage and a baseline cage sampling frequency of 30 s occurring every four cages. The respiratory exchange ratio (RER) was calculated as the ratio of ${\dot{V}}_{CO_{2}}$ to ${\dot{V}}_{O_{2}}$. Locomotion was measured based on beam breaks within a grid of infrared sensors in each cage. Energy expenditure was calculated using the Weir equation (Weir, 1949): Energy expenditure = 3.941 kcal/L × ${\dot{V}}_{O_{2}}$ + 1.106 kcal/L × ${\dot{V}}_{CO_{2}}$. After 48 h, mice were anaesthetized with pentobarbital (Euthansol 120 mg/kg BW, i.p.), euthanized through exsanguination of the heart (Medak et al., 2024) and tissues harvested.

#### 2.4.2 Tissue collection

Mice were anaesthetized with pentobarbital (Euthansol 120 mg/kg BW, i.p.) and euthanized through exsanguination (Medak et al., 2024). Blood was collected via cardiac puncture, immediately placed on ice for ∼30 min prior to centrifugation (1500 *g* for 10 min), and serum was collected. The liver, inguinal white adipose (iWAT), epididymal white adipose tissue (eWAT), gonadal white adipose tissue (gWAT), BAT, hypothalamus and triceps muscles were harvested, snap‐frozen in liquid nitrogen and stored at −80°C until further analysis.

#### 2.4.3 Serum analysis

Metabolites and hormone assays were analysed with the Versamax Tunable Microplate Reader and SoftMax Pro Software (Molecular Devices, San Jose, CA, USA). Insulin (cat. no. 10‐1247‐01, Mercodia Inc., Uppsala, Sweden) and corticosterone (cat. no. 55‐CORMS‐E01, Alpco, Salem, NH, USA) were measured with commercially available enzyme‐linked immunosorbent assay (ELISA) kits. Serum glucose (cat. no. 10009582, Cayman Chemical Co., Ann Arbor, MI, USA), non ‐esterified fatty acids (NEFA) (cat. no. CA97000‐012, Wako Chemicals, Richmond, VA, USA), and β‐Hydroxybutyrate (BHB) (cat. no. 700190, Cayman Chemical Co.) were measured using commercially available colorimetric assay kits.

#### 2.4.4 Real‐time PCR

RNA was extracted from the hypothalamus using TRIzol and Qiagen (Germantown, MD, USA) RNeasy Mini Kits (cat. no. 74106) followed by DNase‐free treatment (Thermo Fisher Scientific, Waltham, MA, USA, cat. no. AM1906) for the removal of genomic DNA. cDNA was synthesized using Superscript II (cat. no. 4368814, Thermo Fisher Scientific), and real‐time PCR was run using SYBR Green Supermix (cat. no. 1725271, Bio‐Rad Laboratories, Hercules, CA, USA) on a Bio‐Rad CFX connect system.

Gene expression was measured relative to the housekeeping genes, *Ppib* in the first experiment or β‐actin (*Actb*) in the second experiment, with the $2^{-\Delta \Delta C_{T}}$ method as previously described. *Actb* and *Ppib* have been shown to be suitable housekeeping genes (Pachot et al., 2004; Sellayah et al., 2008) and we found that these genes were not impacted by menthol or wheel running (*Ppib* sedentary vehicle 21.69 ± 0.18, sedentary menthol 21.54 ± 0.18, voluntary wheel run vehicle 21.65 ± 0.30, voluntary wheel run menthol 21.53 ± 0.16, *Actb* sedentary vehicle 16.75 ± 0.34, sedentary menthol 16.65 ± 0.57, voluntary wheel run vehicle 16.96 ± 0.19, voluntary wheel run menthol 16.83 ± 0.54, raw *C*_T_, means ± SD).

Primer sequences were as follows: *Actb*: rev 5′‐GAGCATAGGCCTCGTAGAT‐3′, fwd 5′‐GACCCAGATCATGTTTGAGA‐3′; *Ppib*: rev 5′‐GCCCGTAGTGCTTCAGCTT‐3′, fwd 5′‐GGAGATGGCACAGGAGGAA‐3′; *AgRP*: rev 5′‐GGTACCTGCTGTCCAAAGCAG‐3′, fwd 5′‐AGTCTGACTGCATGTTGCGT‐3′; *Orexin*: rev 5′‐GTTCGTAGAGACGGCAGGAA‐3′, fwd 5′‐ ACTTTCCTTCTACTACAAAGGTTCCCT‐3′; *Npy*: rev 5′‐TGTCGCAGAGCGGAGTAGTAT‐3′, fwd 5′‐ATGCTAGGTAACAAGCGAATGG‐3′.

#### 2.4.5 Liver glycogen

Glycogen concentrations were measured in liver as described by Schaubroeck et al. (2022). Briefly, tissue was homogenized in 0.5 M NaOH followed by heating at 100°C for 30 min with periodic mixing. Na_2_SO_4_ and ethanol were added to the tissue homogenate, samples were centrifuged at 2000 *g* for 10 min to precipitate glycogen and then resuspended in ddH_2_O. Sulphuric acid and phenol were added to 50 µL of sample and the reaction proceeded for 30 min. Samples were then transferred to a 96‐well plate and absorbance was read at 488 nm.

#### 2.4.6 Statistical analysis

Statistical tests were completed using GraphPad Prism v.10.0 (GraphPad Software, Boston, MA, USA). Body weight gain, body composition, total food intake, glucose area under the curve, tissue weights, and raw *C*_T_ values of housekeeping genes were analysed by two‐way ANOVA. Tukey's *post hoc* analysis was conducted if there was a significant interaction between l‐menthol treatment and voluntary wheel running/treadmill running.

Wheel and treadmill running distances as well as the time spent running until exhaustion, energy expenditure, oxygen consumption, and carbon dioxide emission were analysed using an unpaired Student's *t*‐test, two‐tailed Student's *t*‐test, or non‐parametric Student's *t*‐test when data were not normally distributed. Pearson's correlation coefficient analysis was used to examine associations between serum corticosterone levels and running distance. Homeostatic model assessment of insulin resistance (HOMA‐IR) was calculated based on fasting blood glucose and fasting insulin levels as reported (Bowe et al., 2014). Outliers were identified through Rout's method of regression. Data are presented as means ± SD, and individual data points are shown when possible. A relationship was considered significant when *P* < 0.05.

## 3 RESULTS

### 3.1 Three weeks of topical l‐menthol treatment reduces voluntary wheel running distance in female and male mice

**FIGURE 1:**
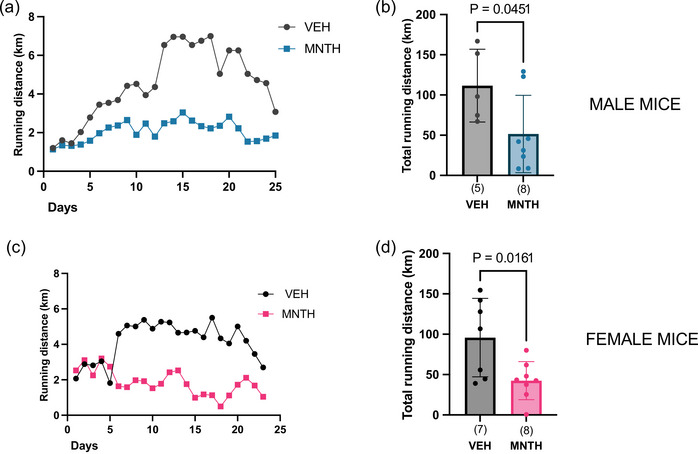
Three weeks of topical l‐menthol treatment reduces voluntary wheel running distance in female and male mice. Three weeks of topical l‐menthol treatments decreased voluntary wheel running distance in male (b) and female mice (d). (a) daily running distance of male mice and (b) total running distance; (c) daily running distance of female mice and (d) total running distance. Data are presented as means ± SD in (c, d) with individual data points shown. Numbers per group are shown in parentheses below each bar. Data were analysed using unpaired, two tailed *t*‐tests in (b) and a Mann–Whitney test in (d). Bars joined by lines are significant at the *P*‐value that is shown.

We were interested in examining the combined effects of menthol treatment and wheel running on weight gain and indices of glucose metabolism in mice. At first, we assessed the effects of menthol treatment on wheel running performance. As shown in Figure 1, daily l‐menthol treatment significantly decreased voluntary wheel running distance in both male mice (*P* = 0.0451) (Figure 1a, b) and female mice (*P* = 0.0161) (Figure 1c, d).

Additionally, the gene expression of neuropeptides linked to wheel running behavior (Landry et al., 2022; MacKay et al., 2019; Ruegsegger et al., 2016; Tesmer et al., 2024) including *Orexin* (exercise: *P* = 0.2085; treatment: *P* = 0.8524; interaction: *P* = 0.0844), *Npy* (exercise: *P* = 0.0864; treatment: *P* = 0.4943; interaction: *P* = 0.6262) and *AgRP* (exercise: *P* = 0.7654; treatment: *P* = 0.8299; interaction: *P* = 0.0855) in the hypothalamus was not different between groups (Table 1).

**TABLE 1:** l‐Menthol does not alter hypothalamic orexigenic neuropeptide gene expression.

	SED VEH	EX VEH	SED MNTH	EX MNTH	*P*
*Orexin*	1.000 ± 0.261(8)	1.291 ± 0.234(7)	1.187 ± 0.320(8)	1.140 ± 0.220(8)	Exercise: 0.2085 Treatment: 0.8524 Interaction: 0.0844
*Npy*	1.000 ± 0.235(8)	1.106 ± 0.246(7)	1.016 ± 0.236(8)	1.204 ± 0.200(8)	Exercise: 0.0864 Treatment: 0.4943 Interaction: 0.6262
*AgRP*	1.000 ± 0.509(8)	1.358 ± 0.643(7)	1.269 ± 0.419(8)	1.014 ± 0.299(8)	Exercise: 0.7654 Treatment: 0.8299 Interaction: 0.0855

*Note*: mRNA expression of *Orexin*, *Npy*, and *AgRP* were analysed in the hypothalmus of female mice after 3 weeks of voluntary wheel running and l‐menthol treatment. Two‐way ANOVA (exercise × treatment) was used to determine differences between groups. Numbers of mice per group are shown in parentheses. Data are presented as means ± SD.

### 3.2 Three weeks of voluntary exercise, but not l‐menthol treatment, improves indices of glucose homeostasis

**FIGURE 2:**
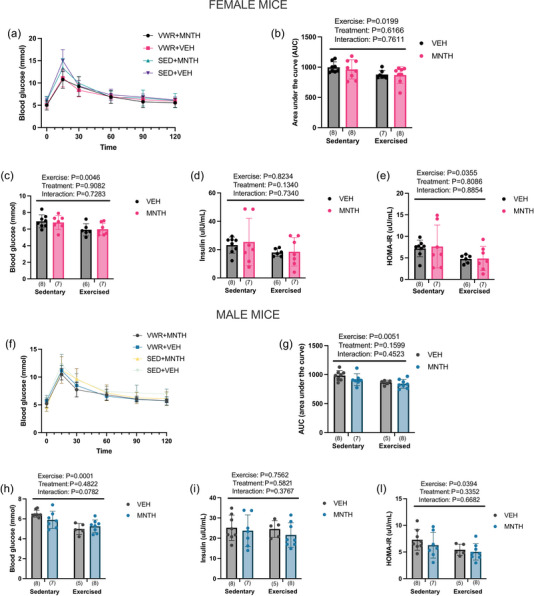
Three weeks of voluntary exercise, but not l‐menthol treatment, improves indices of glucose homeostasis. Three weeks of voluntary wheel running improves glucose tolerance (b, g) and lowers fasting blood glucose (c, h) and HOMA‐IR (e, l) in female and male mice. (a, f) Glucose curves over time during the glucose tolerance test. Two‐way ANOVA (exercise × treatment) was used to determine differences between groups with main and interaction effects shown as an inset. Data are presented as means ± SD with individual data points shown. Numbers per group are shown in parentheses below each bar. HOMA‐IR, homeostasis model assessment‐estimated insulin resistance.

We next aimed to investigate whether l‐menthol would enhance the beneficial effects of exercise on indices of glucose metabolism. We found that daily voluntary wheel running, but not l‐menthol treatment, improved glucose tolerance in both female mice (Figure 2a, b) (exercise: *P* = 0.0199; treatment: *P* = 0.6166; interaction: *P* = 0.7611) and male mice (Figure 2f, g) (exercise: *P* = 0.0051; treatment: *P* = 0.1599; interaction: *P* = 0.4523). Fasting blood glucose was reduced in the exercised female mice (exercise: *P* = 0.0046; treatment: *P* = 0.9082; interaction: *P* = 0.7283) (Figure 2c) and male mice (exercise: *P* = 0.0001; treatment: *P* = 0.4822; interaction: *P* = 0.0782) (Figure 2h). Serum insulin levels were not reduced in either female mice (Figure 2d) (exercise: *P* = 0.8234; treatment: *P* = 0.1340; interaction: *P* = 0.7340) or male mice (Figure 2i) (exercise: *P* = 0.7562; treatment: *P* = 0.5821; interaction *P* = 0.3767) with exercise.

The homeostasis model assessment‐estimated insulin resistance (HOMA‐IR) was reduced in both female (Figure 2e) (exercise *P* = 0.0355; treatment *P* = 0.8086; interaction: *P* = 0.8854) and male (Figure 2l) (exercise *P* = 0.0394; treatment: 0.3352; interaction: *P* = 0.6682) mice with exercise.

### 3.3 Exercise reduces fat mass in female and male mice

**FIGURE 3:**
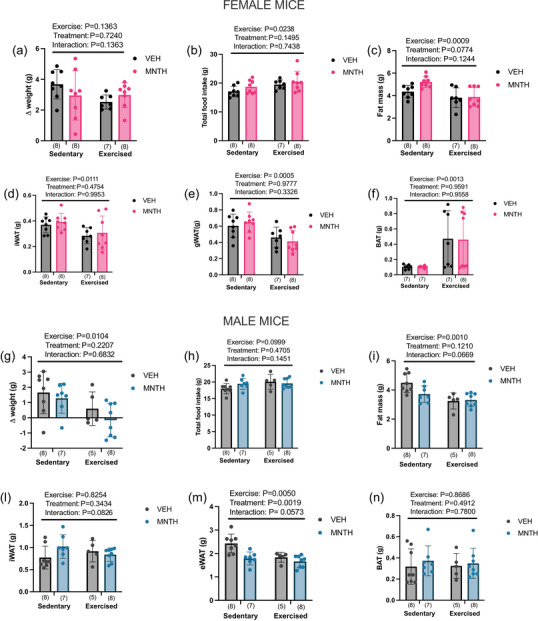
Exercise reduces fat mass in female and male mice. Exercise does not attenuate weight gain (a) but increases food intake (b) in female mice, while attenuating weight gain (g) independent of food intake (h) in male mice. Exercise decreases fat mass in female (c) and male (i) mice and reduces iWAT (d) and gWAT (e) mass in female mice. Menthol and exercise reduce eWAT (m) but not iWAT (l) mass in male mice, while exercise increases BAT mass in female (f) but not in male (n) mice. Two‐way ANOVA (exercise × treatment) was used to determine differences between groups with main and interaction effects shown as an inset. Data are presented as means ± SD with individual data points shown. Numbers per group are shown in parentheses below each bar.

It has previously shown that topical l‐menthol treatments reduce body weight gain in both obese and lean mice housed at thermoneutrality (McKie et al., 2022). In this experiment, exercise did not attenuate weight gain in female mice (Figure 3a) (exercise: *P* = 0.1363; treatment: *P* = 0.7240; interaction: *P* = 0.1363), whereas it attenuated weight gain in male mice (Figure 3g) (exercise: *P* = 0.0104; treatment: *P* = 0.2207; interaction: *P* = 0.6832). Exercise increased total food intake in female mice (Figure 3b) (exercise: *P* = 0.0238; treatment: *P* = 0.1495; interaction: *P* = 0.7438) but not male mice (Figure 3h) (exercise: *P* = 0.0999; treatment: 0.4750; interaction: *P* = 0.1451). Exercise reduced fat mass in female mice (Figure 3c) (exercise: *P* = 0.0009; treatment: *P* = 0.0774; interaction: *P* = 0.1244) and male mice (Figure 3i) (exercise: *P* = 0.0010; treatment: *P* = 0.1210; interaction: *P* = 0.0669). l‐Menthol treatment and exercise reduced eWAT mass (Figure 3 m) (exercise *P* = 0.0050; treatment: *P* = 0.0019; interaction: *P* = 0.0573) but not iWAT mass in male mice (Figure 3l) (exercise: *P* = 0.8254; treatment: *P* = 0.3434; interaction: *P* = 0.0826), whereas in female mice exercise significantly reduced iWAT (Figure 3d) (exercise: *P* = 0.0111; treatment: *P* = 0.4754; interaction: *P* = 0.9953) and gWAT (Figure 3e) mass (exercise: *P* = 0.0005; treatment: *P* = 0.9777; interaction: *P* = 0.3326). Finally, exercise increased BAT mass in female mice (Figure 3f) (exercise: *P* = 0.0013; treatment: *P* = 0.9591; interaction: *P* = 0.9558) but not in male mice (Figure 3n) (exercise: *P* = 0.8686; treatment: *P* = 0.4912; interaction: *P* = 0.7800).

### 3.4 Topical application of l‐menthol increases energy expenditure

**FIGURE 4:**
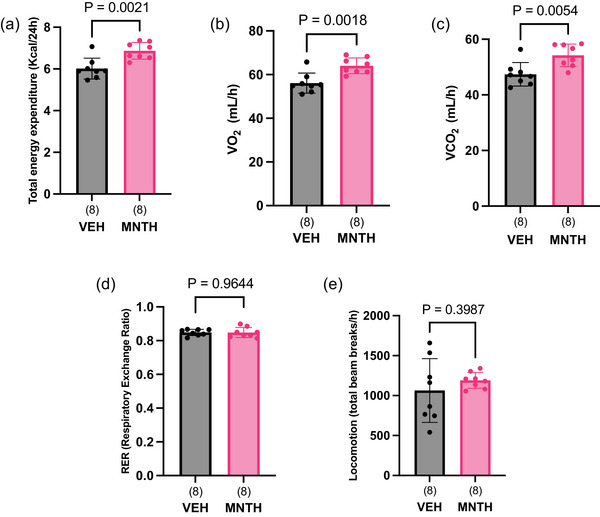
Topical application of l‐menthol increase energy expenditure. Total energy expenditure (a), oxygen consumption (${\dot{V}}_{O_{2}}$) (b), and carbon dioxide production (${\dot{V}}_{CO_{2}}$) (c) are increased in menthol‐treated mice. Respiratory exchange ratio (RER, d) and total locomotion (e) did not differ between vehicle and menthol‐treated mice (d). Data were analysed using unpaired, two tailed *t*‐tests. Data are presented as means ± SD with individual data points shown. Numbers per group are shown in parentheses below each bar.

A previous study demonstrated that acute topical application of l‐menthol increases oxygen consumption, energy expenditure and core body temperature in male mice, through a TRPM8‐dependent mechanism (McKie et al., 2022). We found that l‐menthol treatment increased total energy expenditure (Figure 4a) (*P* = 0.0021), oxygen consumption (Figure 4b) (*P* = 0.0018) and carbon dioxide production (Figure 4C) (*P* = 0.0054), whereas RER (Figure 4d) (*P* = 0.9644) and total locomotion (Figure 4e) *P* = 0.3987) were not affected by l‐menthol treatment in female mice.

### 3.5 Indices of glucose homeostasis are improved following peak wheel running activity and l‐menthol increases serum corticosterone

**FIGURE 5:**
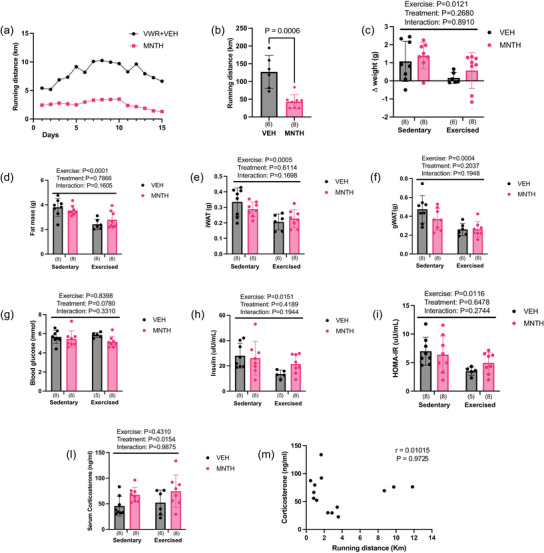
Indices of glucose homeostasis are improved following peak wheel running activity and l‐menthol increases serum corticosterone. Two weeks of l‐menthol treatments reduced running distance (a,b) while exercise decreased weight gain (c), fat mass (d), iWAT (e), and gWAT (f). An acute bout of wheel running decreased insulin levels (h) and HOMA‐IR (i). l‐Menthol increases serum corticosterone (l). Data were analysed using unpaired, two‐tailed *t*‐tests in (b). Two‐way ANOVA (exercise × treatment) was used to determine differences between groups with main and interaction effects shown as an inset. Data are presented as means ± SD with individual data points shown. Numbers per group are shown in parentheses below each bar. HOMA‐IR, homeostasis model assessment‐estimated insulin resistance.

In our initial experiments, endpoints were measured approximately 24 h after the last l‐menthol treatment, with running wheels locked the evening prior. As a next step, we aimed to investigate whether l‐menthol treatment influenced indices of glucose homeostasis when measured immediately following peak wheel running activity. We also sought to determine whether reductions in wheel running due to l‐menthol treatment were reflected in differences in the hypothalamic gene expression of neuropeptides following peak wheel running activity. To do so, we repeated the menthol/wheel running experiment and harvested tissues following peak wheel running activity in the dark cycle (∼00.00 h). These experiments were only completed in female mice as they are better runners than males. We confirmed that topical l‐menthol treatments reduced voluntary wheel running distance (Figure 5a, b) (*P* = 0.0006) and that exercise significantly reduced weight gain (Figure 5c) (exercise: *P* = 0.0121; treatment: *P* = 0.2680; interaction: *P* = 0.8910), fat mass (Figure 5d) (exercise: *P *< 0.0001; treatment: *P* = 0.7866; interaction: *P* = 0.1605), iWAT mass (Figure 5e) (exercise: *P* = 0.0005; treatment: *P* = 0.6114; interaction: *P* = 0.1698) and gWAT mass (Figure 5f) (exercise: *P* = 0.0004; treatment: *P* = 0.2037; interaction: *P* = 0.1948). When they were measured immediately following peak wheel running activity, exercise decreased insulin levels (exercise: *P* = 0.0151; treatment: *P* = 0.4189; interaction: *P* = 0.1944) (Figure 4h) and HOMA‐IR (exercise: *P* = 0.0116; treatment: *P* = 0.6478; interaction: *P* = 0.2744) (Figure 4i) while glucose levels did not differ between groups (exercise: *P* = 0.8398; treatment: *P* = 0.0780; interaction: *P* = 0.3310) (Figure 4g). Furthermore, following peak wheel running activity, we observed increased *Orexin* (exercise: *P* = 0.0247; treatment: *P* = 0.8944; interaction: *P* = 0.5565) and *Npy* (exercise:*P* = 0.0010; treatment: *P* = 0.2777; interaction: *P* = 0.5276) but not *AgRP* (exercise: *P* = 0.0870; treatment: *P* = 0.7175; interaction: *P* = 0.2241) mRNA expression (Table 2). Corticosterone is a stress hormone that increases in proportion to exercise intensity (Girard & Garland, 2002; Isobe et al., 2004) and has previously been shown to reduce voluntary wheel running activity (Singleton & Garland, 2019). We found that 2 weeks of topical l‐menthol treatment increased serum corticosterone levels (Figure 5l) (exercise: *P* = 0.4310; treatment: *P* = 0.0154; interaction: *P* = 0.9875), but serum corticosterone levels did not correlate with the distance run on the wheel (Figure 5m) (*r* = 0.01015; *P* = 0.9725).

**TABLE 2:** Hypothalamic orexigenic neuropeptides gene expression increases following peak wheel running activity.

	SED VEH	EX VEH	SED MNTH	EX MNTH	*P*
*Orexin*	1.000 ± 0.412(8)	1.745 ± 1.182(6)	1.115 ± 0.425(8)	1.563 ± 0.532(7)	Exercise: 0.0247 Treatment: 0.8944 Interaction: 0.5565
*Npy*	1.000 ± 0.357(8)	1.782 ± 0.185(6)	1.313 ± 0.443(7)	1.866 ± 0.703(8)	Exercise: 0.0010 Treatment: 0.2777 Interaction: 0.5276
*AgRP*	1.000 ± 0.464(8)	1.618 ± 0.389(6)	1.329 ± 0.653(7)	1.438 ± 0.611(8)	Exercise: 0.0870 Treatment: 0.7175 Interaction: 0.2241

*Note*: mRNA expression of *Orexin*, *Npy* and *AgRP* were analysed in the hypothalamus of female mice after peak wheel running activity. Two‐way ANOVA (exercise × treatment) was used to determine differences between groups. Numbers of mice per group are shown in parentheses. Data are presented as means ± SD.

### 3.6 Topical l‐menthol treatment does not attenuate distance run or the acute metabolic response to a bout of forced treadmill running

Our first experiments demonstrated that voluntary activity was reduced with l‐menthol treatment. To investigate if this could be due to a physiological limitation, we investigated if l‐menthol reduces treadmill running to a similar extent as it does voluntary wheel running.

**FIGURE 6:**
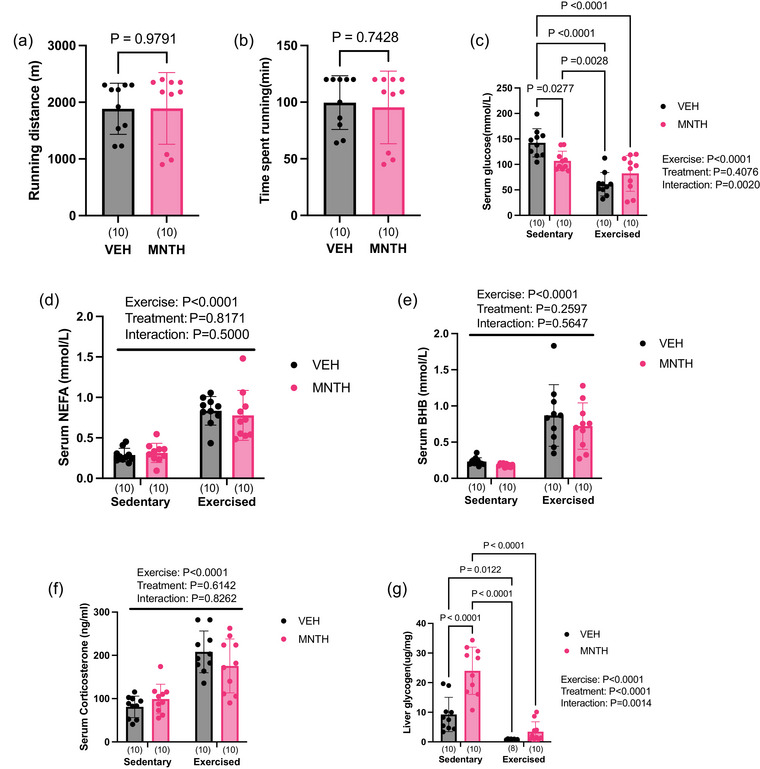
Topical l‐menthol treatment does not attenuate distance run or the acute metabolic response to a bout of forced treadmill running. Treadmill running distance (a) and time to exhaustion (b) are not affected by an acute topical menthol treatment. Exercise reduces circulating levels of serum glucose (c) and increases serum NEFA (d), BHB (e), and corticosterone (f) levels. Exercise and menthol affected liver glycogen levels (g). Data were analysed using unpaired, two tailed *t*‐tests in (a, b). Two‐way ANOVA (exercise × treatment) was used to determine differences between groups with main and interaction effects shown as an inset. Tukey's *post hoc* test was used if a significant (exercise × treatment) interaction was found (c, g). Data are presented as means ± SD with individual data points shown. Bars joined by lines are significantly different at the *P*‐level shown. Numbers per group are shown in parentheses below each bar.

Female mice were treated with either l‐menthol or ethanol control ∼8 h prior to an acute bout of treadmill exercise. We found that l‐menthol had no effect on treadmill running distance (Figure 6a) (*P* = 0.9791), nor did it alter the total time spent running compared to control mice (Figure 6b) (*P* = 0.7428). The acute effects of exercise on circulating metabolites were generally the same for vehicle and menthol‐treated mice. Serum glucose levels were higher in the sedentary vehicle‐treated group compared to the sedentary menthol‐treated group (*P* = 0.0277) (Figure 6c). Additionally, glucose levels were greater in the sedentary vehicle group compared to the exercised vehicle and menthol‐treated groups (*P *< 0.0001) (Figure 6c), and lower in the exercised vehicle‐treated group compared to the sedentary menthol‐treated group (*P* = 0.0028) (Figure 6c). Exercise increased circulating levels of NEFA (Figure 6d) (exercise: *P *< 0.0001; treatment: *P* = 0.8171; interaction: *P* = 0.5000), BHB (Figure 6e) (exercise: *P *< 0.0001; treatment: *P* = 0.2597; interaction: *P* = 0.5647) and corticosterone (Figure 6f) (exercise: *P *< 0.0001; treatment: *P* = 0.6142; interaction: *P* = 0.8262). Liver glycogen levels were significantly lower in the exercised menthol‐treated group compared to the sedentary menthol‐treated group (*P *< 0.0001) (Figure 6g), and in the exercised vehicle‐treated group compared to the sedentary vehicle‐ (*P* = 0.0122) (Figure 6g) and menthol‐treated groups (*P *< 0.0001) (Figure 6g).

We also observed a two‐fold difference in liver glycogen levels between the sedentary menthol and sedentary vehicle‐treated groups (*P *< 0.0001) (Figure 6g). However, liver glycogen levels did not differ between the exercised menthol and exercised vehicle groups (*P* = 0.7500) (Figure 6g).

## 4 DISCUSSION

The aim of this study was to determine whether topical application of the TRPM8 agonist and pharmacological cold‐mimetic l‐menthol would enhance the beneficial effects of voluntary exercise in C57BL/6J mice housed at thermoneutrality. In the current study we found that despite the reduction in running activity with l‐menthol, voluntary exercise improved glucose tolerance, reduced fat mass and blunted weight gain. Similar improvements in indices of glucose metabolism were observed, despite the reductions in wheel running caused by l‐menthol treatment, when assessed immediately following the period of peak wheel running activity. Additionally, the decrease in wheel running with l‐menthol treatment did not appear to be related to changes in hypothalamic neuropeptide gene expression or corticosterone levels. Since l‐menthol reduced voluntary wheel running, we sought to determine whether this could be due to physiological limitations. To do so, we treated mice with l‐menthol and assessed their treadmill running performance. In contrast to wheel running, there were no differences in running distance or time‐to‐exhaustion between l‐menthol‐ and vehicle‐treated mice, and the acute metabolic response to forced treadmill running was essentially the same in both groups. Since l‐menthol treatment did not influence forced exercise, it seems likely that the observed reduction in voluntary wheel running is mediated by a central mechanism that has yet to be fully defined. Supporting this possibility, thermosensory signals, including those from TRPM8, are known to project to brain regions such as the nucleus accumbens, a key centre for motivation and reward‐related behaviour (Ota et al., 2019). This raises the possibility that TRPM8 activation via l‐menthol may modulate exercise behaviour by altering central perceptions of effort or reward. Moving forward, determining the precise neurophysiological mechanisms through which l‐menthol reduces voluntary physical activity needs to be explored in more detail, as does determining if the effects of l‐menthol on wheel running require TRPM8. In regards to the latter point, we have previously shown (McKie et al., 2022) that TRPM8 is required for l‐menthol‐induced increases in energy expenditure, and thus we speculate that it would also be a requirement for reductions in physical activity. Somewhat relatedly, it will also be important to examine whether other TRPM8 agonists, such as icilin, elicit similar effects on physical activity. To date, only one study (Tamura et al., 2019) has investigated TRPM8 activation using icilin in relation to exercise training and found that this compound applied to the hindlimbs of mice subjected to low‐load treadmill stepping led to a preferential recruitment of larger motor neurons.

Our group has previously shown that topical application of l‐menthol increases energy expenditure in sedated mice through a TRPM8 and UCP1 dependent pathway (McKie et al., 2022). Our study extends those findings by showing that menthol similarly increases energy expenditure in freely moving, conscious mice. Notably, despite the reduction in voluntary wheel running due to l‐menthol treatment, the observed increase in energy expenditure could also help explain the exercise‐induced improvements in indices of metabolic health. These findings might suggest that l‐menthol treatment could enable a reduced volume of exercise to be performed to achieve beneficial metabolic effects. Indeed, the magnitude of the training response, such as improvements in endurance capacity, metabolic health and cardiovascular function, can vary based on exercise intensity, frequency and duration (Seo et al., 2014). Given that topical application of l‐menthol has been shown to enhance thermogenesis (Rossato et al., 2014; Valente et al., 2015), improve endurance performance (Tsutsumi et al., 2023) and reduce thermal stress (Mundel & Jones, 2010) during physical exertion (Keringer et al., 2020) in humans, further investigation into its combined effects with exercise training from the standpoint of metabolic health in humans is warranted. In conclusion, our findings showed that while l‐menthol treatment decreased voluntary wheel running distance, exercise still had a significant effect on reducing fat mass, weight gain and improving glucose tolerance. l‐Menthol increased energy expenditure, which may account for the observed improvements in indices of metabolic health, despite a reduction in voluntary exercise.

## AUTHOR CONTRIBUTIONS

AnnaLaura Bellucci and David C. Wright conceived and designed the research. AnnaLaura Bellucci, Bradley J. Baranowski, Stewart Jeromson, Michael Akcan, Serena Trang, Meagan Arbeau, Hadil Alfares, and Katelyn Eisner performed experiments, AnnaLaura Bellucci and Bradley J. Baranowski analysed data. AnnaLaura Bellucci and David C. Wright interpreted results of the experiments. AnnaLaura Bellucci and David C. Wright prepared figures, drafted, and edited the manuscript. All authors approved the final version of the paper. All authors have read and approved the final version of this manuscript and agree to be accountable for all aspects of the work in ensuring that questions related to the accuracy or integrity of any part of the work are appropriately investigated and resolved. All persons designated as authors qualify for authorship, and all those who qualify for authorship are listed.

## CONFLICT OF INTEREST

None declared.

## Data Availability

The curated data are available upon reasonable request
